# St. Bartholomew's Hospital: A Strange Rumour

**Published:** 1903-02-21

**Authors:** 


### Editor's Letter-Box.

[Our Correspondents are reminded that prolixity is a great bar to publication, and that brevity of style and conciseness of statement greatly facilitate early insertion.]

### ST. BARTHOLOMEW'S HOSPITAL: A STRANGE RUMOUR.

F.R.C.S. writes: Intimations have lately appeared that the Committee of Inquiry, nominated on behalf of the Governors of St. Bartholomew's Hospital, by the Lord Mayor and Sir Trevor Lawrence, to investigate and report on certain important questions relative to the future of St. Bartholomew's Hospital, will, among other matters, shortly have under their consideration these two questions: (1) From what districts is the bulk of the patients who attend St. Bartholomew's Hospital derived? (2) Why is the proportion of beds occupied to the total number of beds in the institution so small? It is to be hoped that in their investigation of these matters the Committee of Inquiry will endeavour to make their inquiries cover a long period of time, and will confine their attention entirely to material which existed previously to the Mansion House meeting of January 19th ult. Such a course is rendered advisable, if not absolutely necessary, by the currency of an extraordinary rumour, which is widely prevalent in certain quarters, the truth of which it is difficult to credit, to the effect that instructions have been issued to the house surgeons and house physicians of St. Bartholomew's Hospital, by a responsible official of that institution, to immediately fill up all the vacant beds they possibly can, and at the same time to select for admission as many patients as possible from certain stated districts which are in the close vicinity of that hospital. Whether contradicted or not, such a report is sure to have the effect of diminishing, to a certain extent, public confidence in the impartiality of the investigation now taking place, unless the committee confine their attentions to records and other material which was in existence before the Committee of Inquiry was appointed. It is also essential that immediate reassurances be given to the public by the officials of St. Bartholomew's Hospital, in order that all public doubt upon the matter may be at once removed.

